# FOXN1 Gene Considerations in Severe Combined Immunodeficiency Treatment in Children

**DOI:** 10.7759/cureus.32040

**Published:** 2022-11-30

**Authors:** Stephanie Torres, Michael Marzullo

**Affiliations:** 1 College of Medicine, Edward Via College of Osteopathic Medicine, Monroe, USA; 2 Department of Pediatrics, Christus St. Frances Cabrini Hospital, Alexandria, USA

**Keywords:** scid, rare diseases, pediatrics, severe combined immunodeficiency, foxn1

## Abstract

Forkheadbox N1 (*FOXN1*) gene mutation in humans is a rare cause of thymic hypoplasia and T cell immunodeficiency. This gene is the master transcriptional regulator of thymic epithelial cells and disruptions have been described in consequence to a variety of antepartum complications. *FOXN1* mutation-mediated immune deficiency is typically associated with severe combined immunodeficiency and alopecia universalis (SCID/NUDE phenotypes) with homozygous alterations in human animal models. Less common, however, *FOXN1* alterations can occur in a heterozygous form and provide a distinct phenotype of severe combined immunodeficiency (SCID) without alopecia. Here, we present one such case of a Caucasian child born with heterozygous *FOXN1* mutation, first presenting with undetectable T cell levels at newborn screen. He was confirmed to have *FOXN1* immunodeficiency in the heterozygous form through genetic testing. Early identification and initiation of appropriate interventions are crucial to reduce mortality from opportunistic pathogens associated with immunodeficiency. Furthermore, we need to appreciate the less common presentations of established diseases among young patients.

## Introduction

Severe combined immunodeficiency (SCID) is characterized by low numbers of T cells, natural killer cells, and non-functional B cells. It is a genetically heterogeneous group of diseases with many shared clinical features, constituting the most severe forms of inherited primary immunodeficiency [1]. Screening for SCID is done by measuring thymic function, which is assessed by T cell receptor excision circles (TRECs) as a part of standard newborn screening. This measures T cell output from the thymus. Low levels are suggestive of diminished/absent T cells and must be retested. Standard of care involves follow-up immune system status assays such as mitogen testing and genetic screening. Mitogens are small bioactive proteins or peptides that induce a cell to begin dividing and enhance the rate of mitosis. Mitogen testing measures lymphocyte transformation and function by testing the ability of lymphocytes to proliferate and release cytokines as expected when exposed to these bioactive proteins.

SCID is most well known for being transmitted in an autosomal recessive (AR) or X-linked pattern of inheritance [2]. On genetic testing, our patient returned a heterozygous of unknown inheritance SCID diagnosis. Differential patient response to treatment modalities based on their SCID genotype means that early genetic testing and accurate diagnosis of these patients are critical. A diagnosis of low T/B/NK (natural killer) cell SCID may prompt hematopoietic stem cell transplantation (HSCT) prior to identification of the correct molecular diagnosis. Although the clinical phenotype of classic SCID overlaps with that of *FOXN1* deficiency, definitive diagnosis is critical since thymic transplantation, rather than HSCT, is curative for *FOXN1* deficiency [3]. Furthermore, the main difference in *FOXN1* deficiency defined by homozygous or heterozygous mutation is that heterozygous *FOXN1* mutation patients have the possibility of achieving immunocompetency organically, without the need for transplantation. These patients with partial immune support alone may achieve competency by the age of two to three years. This phenomenon can be explained by laboratory studies which show that although the thymic size and weight are reduced and decreases in the ratio of medullary thymic epithelial cells (mTECs) to cortical TECs (cTECs) is accelerated in mice carrying heterozygous *FOXN1* loss of function mutations similar to what is observed in wild-type mice with aging [4], T lymphocyte development is not impacted [5,6].

## Case presentation

Our patient was an infant male presenting at birth with inconclusive TRECs on the newborn screen and rescreen. Antepartum considerations for the diagnoses of SCID were present in our patient’s medical history. It has been postulated that prematurity and maternal hemolysis, elevated liver enzymes, and low platelet count (HELLP) syndrome are associated with higher rates of SCID and should be worked up [7]. His first encounter within the outpatient pediatric clinic came at four weeks of age after discharge from the neonatal intensive care unit (NICU). Without a formal diagnosis and a failure of communication between the different specialists he was seeing, he was immunized with both live and killed vaccine types until six months of age, which could have had unintended detrimental effects [8]. It was at three months of age that our patient contracted his first opportunistic infection of pneumocystis pneumonia (PCP). During this hospital stay, he further contracted adenovirus infection and Cytomegalovirus (CMV) viremia without CMV retinitis. A profound neutropenia was discovered for the first time on laboratory work during this stay, attributed to the CMV viremia.

At approximately 10 months of age, our patient was officially diagnosed as having several genetic abnormalities (sex chromosome mosaicism) including *FOXN1* heterozygous mutation. The geneticist noted that in patients who have heterozygous mutations T cell numbers typically increase with time and may resolve naturally or require a thymic transplant (Table 1). 

**Table 1:** Clinical notes of patients with compound heterozygous or monoallelic mutations in FOXN1 Table displaying clinical notes and genetic mutations of documented patients with compound heterozygous or monoallelic mutations in *FOXN1*. Patient 1 on the chart is the child in whom we covered in our report. Patients 2-18 are children encountered through the literature and their outcomes are documented exactly as in their respective paper. *FOXN1*: Forkheadbox N1 IVIG: Intravenous immunoglobulin

Clinical notes of patients with compound heterozygous or monoallelic mutations in FOXN1			
Patient	DNA mutation	Clinical notes	Reference
1	FOXN1 Heterozygous unknown allelic variant at this time	Multiple viral infections, IVIG + prophylaxis, self stabilizing T cell count with age	
2	C.933_93dupACCC, c.1089_1103del15	Multiple viral infections, death at 1 year from parainfluenza virus	[9]
3	c.1288C>T, c.1465delC	Bone marrow transplant, healthy with no recurrent infections, on IVIG	[9]
4	c.1465delC	Healthy no recurrent infections	[9]
5	c.1465delC	Healthy no recurrent infections	[9]
6	724C>T	Multiple infections, otherwise healthy	[9]
7	958C>T	Healthy no recurrent infections	[9]
8	962A>G	Death from coronavirus encephalitis in infancy	[9]
9	982T>C	Healthy no recurrent infections	[9]
10	1075G>A	Healthy no recurrent infections	[9]
11	1201_1206del16	Healthy no recurrent infections	[9]
12	1201_1206del16	Healthy no recurrent infections	[9]
13	1201_1206del16	Healthy no recurrent infections	[9]
14	1201_1206del16	Healthy no recurrent infections	[9]
15	1201_1206del16	Healthy no recurrent infections	[9]
16	1293delC	Remaining low T count prophylaxis continued for life	[9]
17	1418delC	Remaining low T count prophylaxis continued for life	[9]
18	Copy number variation of unknown segment	Multiple viral infections, death at four months of age	[10]

At this time, our patient’s T cell numbers were still low but progressively improving. He was still testing positive for CMV infectivity after early infection at three months of age. The plan was to do broad prophylaxis and intravenous immunoglobulin (IVIG), with reconstitution panels drawn every two to three months. Our patient was placed on prophylactic azithromycin, IVIG, valganciclovir, voriconazole, amoxicillin, cytomegalovirus immune globulin and pentamidine; with granulocyte colony-stimulating factor (G-CSF), and Imm glob (Ig), gam(IgG)-gly-IgA 0-50 as stimulatory agents for immune regeneration. It was noted that the patient could wean off medication prophylaxis as T cell proliferation studies became acceptable (mitogen/antigen testing and mayo T cell panel) and naïve T cell count reaches 200/ul or above.

As T cell count rose with age, our patient was able to scale back his prophylaxis to palivizumab for respiratory syncytial virus (RSV) prevention due to abnormally high RSV transmission that calendar year, IVIG, and sulfamethoxazole-trimethoprim, with granulocyte colony-stimulating factor (G-CSF), and Ig, gam(IgG)-gly-IgA 0-50 as stimulatory agents for continued immune regeneration. Mitogen response was tested to ensure that T cells could be stimulated but antigen response remained low indicating that the T cells can be stimulated to proliferate but do not respond well to things they have seen previously through the release of cytokines.

At 15 months of age, naïve T cell count was 220/ul, up from 140/ul at 12 months of age, making our patient above the SCID range which is <200/ul. All these values were obtained via flow cytometry on monthly screens of T cell proliferation studies of mitogen/antigen testing and a Mayo T cell panel.

At the time of submission, the patient is 22 months of age and has progressed to only requiring sulfamethoxazole-trimethoprim. He is developmentally within normal limits and continuing to stabilize his T cell counts with the aid of stimulatory agents.

## Discussion

The *FOXN1* gene contains eight coding exons with a DNA binding domain in exons 6-7 and a transactivation domain between exons 8-9. Whether the mutation is homozygous or heterozygous seems to be related to whether impacts are found on the DNA binding or transactivation domains of *FOXN1* [9]. Not commonly done, nor verified for reliability in larger patient populations, exome sequencing determines every letter in a DNA sequence, not only the ones known to vary, so it can reveal rare mutations that genome-wide association study (GWAS) would not uncover. Exome sequencing is a good choice for uncovering rare mutations. Despite advances in newborn screening guidelines and genetic testing, there are still children misdiagnosed or simply missed when it comes to more rare variants within the SCID family of disease [10]. Moreover, genetic counseling for families relies on accurate decoding of the diagnosis in question [11]. It is these kinds of cases which bring to light the need for more attention to mapping of potential, though little known, genetic variants of established disease.

As previously stated, treatment options differ depending on the genetic variant of disease. Whether a child will most benefit from HSCT, thymic transplantation, or medical management lies within their genetic blueprint. The HSCT that is curative in a non-*FOXN1* SCID child could lead to unnecessary surgical complications or even death [12] in *FOXN1* heterozygous SCID children. Thymic transplantation has been shown to generate tumors with all the characteristics of T cell acute lymphoblastic leukemia (T-ALL) [13]. Taken together, knowing the genetic variant of the patient in question can be the key to avoiding unnecessary surgical complications or the use of scarce resources.

Many studies have demonstrated that children with primary immunodeficiencies have the greatest mortality within the first year of life [14]. Our patient was at the 10-month mark when a diagnosis was made, and an actionable plan could be implemented. Most of this time was spent, unfortunately, waiting for appointments with specialists, which in less urban areas were difficult to find due to shortage of such physicians. This speaks volumes about the current physician shortage in the United States, especially within less population dense regions [15]. We can continue to expect poor outcomes for patients who cannot access care within reasonable distances from their homes.

Lastly, there is a role to be played by maternal healthcare improvement given that the transfer of maternal antibodies has long been recognized as a central component of newborn immunity against pathogens [16]. The United States maternal mortality rate has climbed year to year from 2018-2020. As per the CDC (Centers for Disease Control and Prevention), the 2020 maternal mortality rate was 23.8 per 100,000 live births which is more than double that of most other high-income countries [17]. If we are to prevent primary immunodeficiencies, or any other form of inheritable disease linked to poor maternal health, then it behooves us as a nation to put more funding and attention toward the health of women during their reproductive years. 

## Conclusions

To investigate the nuances and best practices between different SCID phenotypes in children, patients reporting low or inconclusive TRECs on repeat neonatal screens should be carefully monitored and immediately connected to genetic testing services in order to avoid delay in targeted treatment. Key symptoms to differentiate patients with varying types of SCID include lymphopenia, leukopenia, agammaglobulinemia, neutropenia and the presence or absence of alopecia and nail dystrophy. Ultimately, patients do differ from textbook presentations and exist along a spectrum of *FOXN1* or alternate mutations and thus would most benefit from timely genetic testing. Further investigation of the relationship between the spectrum of genotypes representing SCID and response to first-line treatments is warranted to identify the possible causality in outcomes. 
